# Holobionts and their hologenomes: Evolution with mixed modes of inheritance

**DOI:** 10.1590/1678-4685-GMB-2017-0070

**Published:** 2018-03-01

**Authors:** Karen Luisa Haag

**Affiliations:** Departamento de Genética, Instituto de Biociências, Universidade Federal do Rio Grande do Sul (UFRGS), Porto Alegre, RS, Brazil

**Keywords:** Genomics, metagenomics, microbiota, symbiosis, holobiont

## Abstract

Symbioses are ubiquitous and have played an influential role in the evolution of life on Earth. Genomic studies are now revealing a huge diversity of associations among hosts and their microbiotas, allowing us to characterize their complex ecological and evolutionary dynamics. The different transmission modes and the asynchronous cell proliferation of the numerous symbionts associated with one host generate a genomic conflict ought to be solved. Two disputing views have been used to model and predict the outcome of such conflicts. The traditional view is based on community ecology, and considers that selection at the level of individuals is sufficient to explain longstanding associations among species. A new perspective considers that the host and its associated microbiota constitute a biological entity called holobiont, and that regarding it as a higher-level unit of selection is unavoidable to understand phenotypic evolution. Novel extended phenotypes are often built through symbiotic interactions, allowing the holobiont to explore and survive in distinct environmental conditions, and may evolve in a Lamarckian fashion.

## The hologenome concept and its roots

You are what you eat, what you live on, what lives on you, and what lives in you (Keeling and Palmer, 2008). The idea that organisms do not evolve independently, but rather in conjunction with all their associated symbionts traces back to writings from the end of the 19^th^ century by Russian evolutionary biologists (see, for example, Mereschkowsky, 1905). ‘Symbiogenesis’, the evolutionary origin of biological innovations by sybmbiosis, reinforces the role of admixture and hybridization in evolution, rather then isolation and dichotomy (Margulis and Fester, 1991; Khakhina, 1992). The application of genomic tools revealed that all living organisms harbor large and diverse assemblages of symbiotic microorganisms, challenging the common view in which species are the major evolutionary units (Doolittle and Zhaxybayeva, 2010). Recent estimates confirm that bacterial cells outnumber, or at the very least, equal the amount host cells in the human body (Sender *et al.*, 2016). Microbes sustain life on our planet exactly because of their myriad associations and biogeochemical processes; although gnotobiotic animal life is possible inside a bubble, such a condition is known to have deleterious effects on the development of both immune and nervous systems (Gilbert and Neufeld, 2014; Mayer *et al.*, 2015). Most mechanisms by which symbionts influence the metabolism, physiology, immunity, behavior and development of their hosts are yet to be discovered. Similarly, the evolutionary mechanisms underlying the evolution of symbiotic assemblages remain elusive.

Traditionally, evolutionary biologists have viewed changes within individual genomes as the major source of phenotypic variation leading to adaptation through natural selection, and ultimately generating diversity among species. Mathematical models describing the evolution of symbioses have focused on a restricted number of interacting partners evolving by natural selection at the level of individuals (Yamamura, 1996; Genkai-Kato and Yamamura, 1999; Morris *et al.*, 2012). The ‘holobiont’, referring to the host and its associated microbiota (Mindell, 1992), and its ‘hologenome’ i.e., nuclear, organelle and microbiome genomes (Zilber-Rosenberg and Rosenberg, 2008), provide a broader modeling framework by capturing the integrative nature of host-microbiota associations, and demand multilevel selection as well as inheritance of acquired characteristics (Liu, 2011; Bordenstein and Theis, 2015). Thus, theoretical models aiming to characterize holobiont evolution should account for the genome conflicts generated by distinct modes of transmission of microbiota members.

## Microbiota transmission and holobiont cohesion

**Figure 1:**
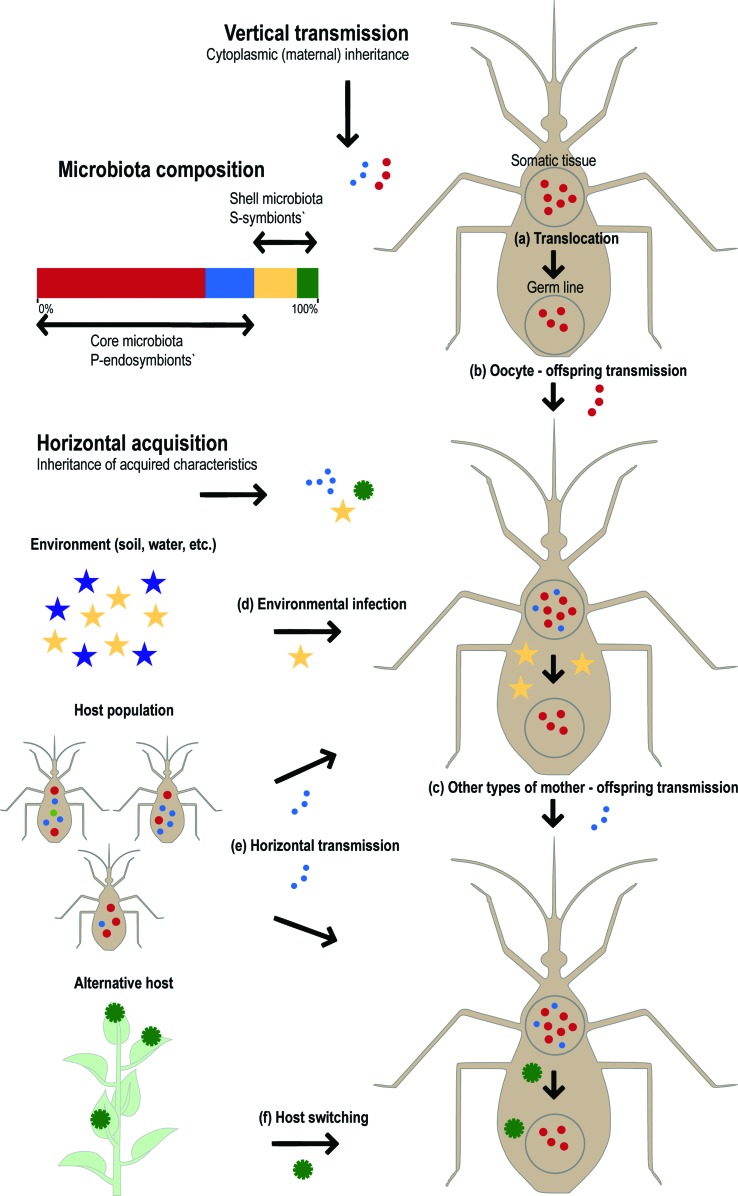
Modes of microbiota transmission and its bi-layered composition. Vertical transmission is accomplished by the translocation of symbionts from the somatic to the germline tissue (a). Generally the females, which contribute with their oocyte cytoplasm to the zygote, are able to vertically transmit P-endosymbionts, or core microbiota, to their progeny (b). There are numerous other behavioral or physiologic mechanisms by which vertical transmission of P- or S-symbionts might be accomplished (c; reviewed in Funkhouser and Bordenstein, 2013). Both the core and shell microbiota might be horizontally transmitted. Whereas facultative S-symbionts are usually acquired from the environment and might be lost in subsequent generations (d), core symbionts that are acquired from the environment or from other members of the population (e) must be regularly horizontally transmitted or engage some form of vertical transmission to remain in the host population. Symbionts may also switch among distantly related hosts (f) as part of their life cycles or as an opportunistic strategy. Colors of the bar column representing microbiota composition correspond to hypothetical symbionts with distinct modes of transmission as depicted in the figure.

The holobiont is a complex community that maintains its cohesion by vertical transmission - or recurrent horizontal acquisition - of a stable microbiota on the one hand, and on the other hand is open to the acquisition of novel microbiota members through horizontal transmission, environmental infection, or host switching (Figure 1). Most symbionts, including mutualists, commensals, parasites and pathogens, have mixed modes of transmission (both vertical and horizontal; Ebert, 2013), which combined with their environmental gain, or random loss, leads to a dynamic pattern of holobiont composition across generations. Nonetheless, hosts with greater genetic divergence may still exhibit more distinguishable microbiota, a pattern called “phylosymbiosis” (Brucker and Bordenstein, 2013; Brooks *et al.*, 2016).

How do symbionts and their hosts establish specific, longstanding associations, without vertical transmission? By characterizing the progression of bacterial colonization of *Hydra* polyps at various time points and modeling the organizational principles of this colonization process, it was found that the colonization rate depended on local environmental or host-derived factors as well as interactions between individual bacteria (Franzenburg *et al.*, 2013). Such findings could be extended to the process of microbial gut colonization in metazoans, which seems to involve many of the same host factors that are usually involved in attacking bacteria (for example, Toll-like receptors and immunoglobulins), but symbiotic bacteria seem to have certain compounds on their surface that turn this recognition into acceptance rather than attack (Chu and Mazmanian, 2013). Therefore, phylosymbiosis is not necessarily due to coevolution, since it does not imply that members of the microbial community are constant, stable, or vertically transmitted from generation to generation (Brooks *et al.*, 2016).

To understand how the microbiota structure evolves, a rather simplified bi-layered model of microbial composition is normally used (Shapira, 2016), reflecting more or less how tight are the symbiotic associations within the holobiont. Whereas the ‘core’ microbiota is stable in the host population and is normally composed by a limited number of host-specific primary endosymbionts (P-symbionts; Moya *et al.*, 2008) that may evolve mutualistic interactions with their hosts, the ‘shell’ microbiota, which includes secondary facultative symbionts (S-symbionts), is a consequence of shared host-symbiont environment, and may not endure (Figure 1). However, the definition of a core microbiota is not straightforward; it should be taken as a statistical reality that is relative to the sampling effort and to the environment and does not always express the degree of host-symbiont intimacy (Ebert and Qi, 2011). Furthermore, symbionts shifted from an S- to a P-symbiotic lifestyle multiple times in their evolutionary history (Nováková *et al.*, 2009), implying that the distinction between core and shell may change in time as well. Interestingly, the distinction between core and shell is not strictly associated with the symbiont mode of transmission.

Classic examples of vertically transmitted obligate mutualists of the core microbiota are bacteriocyte-associated endosymbionts of plant sap-sucking insects (Figure 1a), such as the gamma-proteobacterium *Buchnera aphidicola* in aphids (reviewed in Baumann, 2005). Because symbionts are harbored inside a specialized morphological structure, the bacteriocyte, within all (or most) of the insects of a taxonomic group, and since the symbiont is essential for the survival of the host, it is assumed that such associations result from a single ancient infection of an insect. Indeed, the Buchnera-aphid association is estimated to be as old as 230 mya (Ochman *et al.*, 1999). Nevertheless, the core microbiota may also include host-specific symbionts that are recurrently acquired from the environment (Figure 1d), such as the luminous bacterial mutualist *Vibrio fischeri* of the squid *Euprymna scolopes* (Nyholm and McFall-Ngai, 2004), or transmitted as an assemblage by other members of the host population (Figure 1e), such as the Gram-negative bacteria that belong to the core honey bee microbiota (Powell *et al.*, 2014). The shell microbiota generally includes free-living opportunistic microorganisms that are common in the environment and may become beneficial or pathogenic. For example, different species of *Trichoderma* are found free in the soil, but may also associate to plant leaves and roots, protecting them against pathogenic fungi, or even parasitize other fungi (Harman *et al.*, 2004). Other opportunistic fungi include commensal organisms such as different species of *Candida* that colonize distinct body locations in humans, in particular the gastro-intestinal tract, the genital tract, or the skin, where they may become pathogenic (d’Enfert, 2009). Shell symbionts may also be acquired by host switching, and jump between hosts as distantly related as plants to insects, e.g., *Candidatus* Phytoplasma, a Gram-positive bacterium that has diverse pathogenic effects on different plant species and is mostly harmless on the insect vectors (Hogenhout *et al.*, 2008).

Holobiont dynamics and evolution is based upon the interactions among all symbionts and their host. The environmental acquisition of a new symbiont may have profound effects on the entire community structure. For example, after ingesting a blood meal containing *Trypanosoma cruzi* epimastigotes, the diversity of the gut microbiota of the triatomine vector increases, mediated by the insect immune responses (Díaz *et al.*, 2016). In bumble bees it was shown that gut symbionts may even impede the establishment of an immigrant, protecting the holobiont against pathogenic trypanosomes (Koch and Schmid-Hempel, 2012), though this protection seems to rely on a balanced microbiota composition. Some honey bee gut symbionts such as the beta-proteobacterium *Snodgrassella alvi* are known to protect against trypanosomes, but young bees experimentally fed with additional *S. alvi* are counterintuitively more susceptible to the parasite (Schwarz *et al.*, 2016).

The complex holobiont dynamics, in addition to the plethora of ways by which it might be disturbed, have lead to numerous criticisms of the holobiont as a unit of selection (Moran and Sloan, 2015; Douglas and Werren, 2016). However, holobiont cohesion might depend less on symbiont transmission modes, or on their shared interests, and more on the specificities of (symbiont)^n^ x host interactions and their respective population histories. Associations between host and symbiont genes can be described by the same dynamic model as conventional linkage disequilibria between genes in the same genome, and covariance between host and symbiont genomes depends on geographic structure, selection, and co-transmission rate (Fitzpatrick, 2014).

## Genetic inheritance and hologenomic conflict

‘Genomic conflicts’, which refer to a dispute of interests caused by the different modes of proliferation and inheritance of distinct genomic segments (Trivers and Burt, 2009) are not confined to symbiotic assemblages. They are found in every replicating eukaryotic cell, i.e., in addition to the classical parent-offspring Mendelian inheritance of nuclear genes that replicate synchronously during mitosis or meiosis, there are various other ways by which genes within one cell are multiplied and transmitted. An individual could be considered to be the product of a successful mutualism of its constituent genes, but sexual reproduction, based on the union of gametes with different genomes, underlies many conflicts, providing conditions for the spread of alleles that help to reduce the competition between different mating types. The fact that most sexual species only have two mating types is intriguing. One hypothesis states that selection at the level of individuals may have favored the spread of nuclear genes that coordinate the inheritance of cytoplasmic genomes - enforcing uniparental inheritance - preventing the competition between unrelated cytoplasmic genomes (Hurst, 1995). Conflicts can even be found within the realm of a single genome. Thus in many species, including humans, more than half of the genome is derived from selfishly replicating transposons (TEs; Koning *et al.*, 2011). The skewed distribution of TEs in most species results from an interplay between evolutionary forces countering TE expansion and host epigenetic transposon silencing mechanisms that evolved by natural selection (Hollister and Gaut 2009). Even though most transposition activity is associated with detrimental phenotypic effects, there are numerous examples of adaptations conferred by TEs that have been domesticated, such as the industrial melanism in *Biston betularia* (Hof *et al.*, 2016). Furthermore, TEs may have the potential to provide host genomes with the ability to enhance their own evolution (Kidwell and Lisch, 2000).

Besides moving within a single genome, TEs have also the propensity to move across genomes of different species by horizontal gene transfer (HGT; Silva *et al.*, 2004). In eukaryotes, HGT may be accomplished through various routes, ranging from species hybridization (Scavariello *et al.*, 2017) to symbiosis (Schaack *et al.*, 2010). Among prokaryotes HGT seems to be common - estimates based on comparative genomics suggest that up to 15% of an entire prokaryotic genome might be derived from HGT (Koonin *et al.*, 2001) - and is thought to be a replacement for sex to avoid Muller’s ratchet (Koonin, 2011). The utter importance of HGT for bacteria is such, that specialized viral-like particles produced by alpha-proteobacteria called GTAs (Gene Transfer Agents) exist to mediate HGT in coastal and oceanic environments (McDaniel *et al.*, 2010). Not surprisingly, symbionts and their hosts are common HGT players, e.g., parasites may co-opt host genes for their own benefit (Pombert *et al.*, 2015) or vice-versa (Mower *et al.*, 2004). Major transitions in the evolution of life, such as the origin of eukaryotes, had HGT at their roots (Koonin, 2016), and HGT certainly continues to shape how our genomes are and what they do (Koonin *et al.*, 2001; Keeling and Palmer, 2008).

## The holobiont as a unit of selection

Natural selection, increasing the population frequency of beneficial genes, and purging those with detrimental effects, is one of the central principles of current evolutionary theory and explains how genomic conflicts are solved at the level of individuals. Selection at higher levels, such as smaller groups within a population (see for example, Wilson, 1975) or species within a clade (for example, Stanley, 1975), have been viewed with skepticism. The common denominator of all levels of selection is differential survival or reproduction of an inherited feature expressed in a phenotype, no matter where it is manifested, in an individual, population, species, or even a community. Phenotypes are not always reduced to individuals but may constitute a feature that is shared by a group of individuals in the form of ‘extended phenotypes’ (Dawkins, 1978). Because holobionts essentially do not differ from individuals, neither regarding genomic conflicts, nor with respect to their basic genetic mechanisms of inheritance (Table 1), it is logical to assume that their extended phenotypes are amenable to selection. Analogous to transposable elements and organellar genomes of the host, symbiont genomes within the hologenome can be transmitted horizontally and/or vertically, replicate independently from each other and from the host genome, generating a ‘hologenomic conflict’. Moreover, phenotypic novelty might be introduced to the holobiont by a new colonizing symbiont.

**Table 1:** Examples of genetic transmission modes that apply both to individuals and holobionts referred in this study.

	Individual	References	Holobiont	References
Vertical inheritance	Uniparental inheritance of cytoplasmic genomes	Several examples are described in Hurst (1995)	Vertical transmission of endosymbionts	Reviewed in Baumann (2005); Fukatsu and Hosokawa (2002) show an example of transmission via symbiont capsule in stinkbugs; Hoffmann *et al.* (2011) show an application in the control of mosquito vectorial competence; Mira and Moran (2002) describe *Buchnera* population bottlencks after their vertical transmission in aphids; Ochman *et al.* (1999) find that the rates of molecular evolution in vertically transmitted endosymbionts is ramarkably similar.
Horizontal inheritance	Horizontal gene transfer (HGT)	Keeling and Palmer (2008) and Schaack *et al* (2010) review HGT in eukaryotes; Koonin *et al* (2001) review HGT in prokaryotes; Koonin (2016) discusses the roles of HGT in major evolutionary transitions; McDaniel *et al.* (2010) estimate the rate of HGT mediated by GTAs in the oceans; Pombert *et al* (2015) show an example of host to parasite HGT.	Horizontal transmission of symbionts	d’Enfert (2009) reviews the survival strategies of *Candida*; Franzenburg *et al.* (2013) describe the developmental pattern of microbiota colonization in *Hydra*; Geissbühler *et al.* (2009) shows an application that kills mosquito vectors; Harman *et al.* (2004) review the biology of *Thichoderma* opportunistic symbionts of plants; Hogenhout *et al* (2008) review the biology of opportunistic phytoplasma symbionts of plants and insects; Janson *et al.* (2010) show the evolutionary pattern of gall forming fungi harvested and transmitted by midges; Nyholm and McFall-Ngai (2004) review the squid-vibrio symbiosis responsible for bioluminescence; Powell *et al* (2014) characterize the developmental pattern of microbiota colonization of the honeybee gut.
Vertical and horizontal inheritance	Transposable elements (TEs)	Hof *et al.* (2016) shows that the mutation causing melanism in British peppered moths is caused by a TE; Hollister and Gaut (2009) characterize the interplay between epigenetic and evolutionary forces against TE expansion; Kidwell and Lisch (2000) review the evidence of beneficial effects of TEs on host genomes; Scavariello *et al.* (2017) show an example of TE HGT in insects; Silva *et al.* (2004) characterize factors involved in the HGT of TEs.	Mixed modes of symbiont transmission	Díaz *et al.* (2016) demonstrate that the infection of triatomines with *Trypanosoma cruzi* mostly affect the environmentally acquired insect symbionts; Ebert (2013) provides an extensive review of symbioses with mixed modes of transmission; Koch and Schmid-Hempel (2012) show that gut microbiota of bumble bees can account for the specificity of interactions between the insect and tripanosomatids; Nováková *et al.* (2009) describe the evolution of *Arsenophonus*, a highly diverse clade of symbionts with heterogeneous life styles.
Inheritance of acquired characteristics	Epigenetic inheritance and some cases of HGT	Jablonka and Lamb (1999) provide an extensive review of epigenetic inheritance arguing that it constitues a case of Lamarckian evolution; Koonin and Wolf (2009) define what they call quasi-Lamarckian, Lamarckian and Darwinian evolution.	New, non-random (adaptive) symbiont acquisition	Brown *et al.* (2014) suggest that stinkbugs adapted to a new foodplant through the acquisition of a new symbiont by host switching.

Vertically transmitted genes from obligatory mutualists that are harbored in specialized host cells called bacteriocytes need to be translocated into oocytes (Figure 1a). Their transgenerational dynamics is similar to organellar genomes, but their evolution may suffer a stronger effect of genetic drift, due to recurrent bottlenecks caused by translocations (Mira and Moran, 2002). Horizontally inherited symbiont genomes, on the other hand, may be less affected by drift if maintained in large reservoir populations in the environment. It remains to be investigated how the evolutionary factors acting at the level of individuals within symbiont populations interact with the higher-level factors of the holobiont. Intuitively it is expected that genomes of symbionts may proliferate selfishly, compete with other symbiont genomes and end up being eliminated, or being acquired and become incorporated into the hologenome and maintained. Being able model how these selection pressures acting at different holobiont levels interact, based on their directions and intensities, would have numerous applications, particularly in the manipulation of vectorial competence.

Vector-borne diseases are caused by pathogens that must overcome the immune responses elicited by the insect gut microbiota. Symbionts can also directly impair pathogen infectivity or viability, independent of the host, or influence pathogen transmission by altering the host life history (van Tol and Dimopoulos, 2016). Several microbiota-based disease control strategies have been developed for the mosquito vector, including its infection with *Wolbachia* for dengue control (Hoffmann *et al.*, 2011) and infection with entomopathogenic bacteria such as *Bacillus thuringiensis* and *B. sphaericus* that kill mosquito larvae, reducing transmission of malaria (Geissbühler *et al.*, 2009). In paratransgenesis, a mosquito symbiont is genetically manipulated to express a factor that hinders vector competence (Wang and Jacobs-Lorena, 2013). However, to ensure the long-term viability of such strategies, in face of ongoing evolutionary change, the interaction of distinct holobiont selection pressures needs to be dissected.

## Inheritance of characters acquired through symbiosis

The hologenome is a vehicle for the inheritance of acquired characters, because symbiont-induced phenotypes may be adaptive to the host. Let us imagine a gene that is adaptive in a certain environment, e.g., a bacterial gene that allows metabolizing a substance that is common in the environment and is otherwise toxic. The acquisition of such a bacterial symbiont by another organism would allow its survival in such an environment. An advantage induced in an organism by an external (environmental) source, and which is maintained in further generations, is considered ‘Lamarckian’ evolution, as opposed to the ‘Darwinian’ form of evolution, which is based on the advantage of random mutations (Jablonka and Lamb, 1999; Koonin and Wolf, 2009). In the context of the hologenome, it is easier to understand adaptations by regarding it as a higher-level unit of selection. I will illustrate this by changes in insect life history traits induced by their symbionts.

Phytophagous stinkbugs from the genus *Megacopta* maintain nutritional symbionts - the extracellular gamma-proteobacterium symbiont ‘*Candidatus* Ishikawaella capsulata’ - in the cavity of crypt-bearing posterior midgut, which are vertically transmitted due to a unique translocation mechanism called ‘symbiont capsule’. Newly hatched nymphs use their piercing and sucking mouthparts to probe for symbiont-containing capsules laid down with the eggs by their mothers (Fukatsu and Hosokawa 2002). It was shown that the pest status of these insects is principally determined by symbiont genotype rather than by insect genotype. A pest stinkbug species, *Megacopta punctatissima*, performs well on crop legumes, while a closely related non-pest species, *M. cribraria*, suffers low egg hatch rate on the same plants. When their obligate gut symbiotic bacteria are experimentally exchanged between the species, their performance on the crop legumes is reversed: the pest species shows low egg hatch rate, whereas the non-pest species restores its normal egg hatch rate (Hosokawa *et al.*, 2007). Genomic analyses suggest that the invasion of formerly wild-plant-adapted stinkbugs onto soybean in the US possibly occurred via host switching of the symbiont from unrelated soy-adapted stinkbugs (Brown *et al.*, 2014). Host switching is a likely explanation in this case, because when nymphs are disturbed or capsules are damaged or not found, nymphs rapidly disperse in search of other capsules (Hosokawa *et al.*, 2008). Lamarckian evolution is implicated, because the ability to feed on the crop plant is acquired from the environment, not by random mutations. Furthermore, it is the holobiont, with its hologenome, that evolves via an extended phenotype conferred by the symbiont.

The case above involves a vertically transmitted symbiont that strictly coevolves with the host (Hosokawa *et al.*, 2006). However, neither vertical transmission, nor coevolution is required for hologenome evolution. An interesting example comes from the ectosymbiotic association of the fungus *Botryosphaeria dothidea* that is harvested by the midge *Asteromyia carbonifera* inside galls of the host plant *Solidago altissima.* Striking gall morphologic variation is found sympatrically (in the same habitat) and syntopically (on the same host plant; Crego *et al.*, 1990). Although galls result from the growth of the fungus inside the plant tissue, it is known that gall variation does not derive from the fungus, but rather seems to result from midge ‘manipulation’. Genetic studies show that midges from distinct gall morphs are highly differentiated (Stireman *et al.*, 2008), though there is no evidence of gall morph-associated phylogenetic, genetic or phenotypic divergence in the symbiont: there is also no evidence of genomic correlates of a symbiotic lifestyle for the bacterium and essentially no evidence of evolutionary divergence of the symbiotic isolates (midge associated) from free-living (not insect associated) *B. dothidea* populations (Janson *et al.*, 2010). Vertical transmission and reciprocal changes between the fungus and the midge are not implicated in gall morphologic evolution in this association, probably because the fungus is recurrently acquired from the environment and slaved by the midge. Extended phenotypes emanating from ecological associations may have strong impacts in community ecology and evolution, suggesting that an integration of ecology and genetics is probably essential for understanding the natural world (Whitham *et al.*, 2003).

## Conclusion: why do we need a hologenome?

The hologenome perspective helps us to pursue the causes of phenotypic variation and evolution. Viewing the holobiont as a unit of selection - not a superorganism - allows understanding that in a complex community of interacting organisms there are emergent (extended) phenotypes. Symbionts are drivers of the evolution of their hosts. They are known to alter the reproduction system and may influence the mating preferences of their hosts, leading to reproductive isolation and speciation (Sharon *et al.*, 2010; Shropshire and Bordenstein, 2016). Symbiont genes may also allow their hosts to explore new environments through extended phenotypes. Invasive species such as sap-feeding insects have to rely on their bacterial symbionts to spread on new host plants (Brown *et al.*, 2014). Similarly, vectorial competence of insects that transmit severe diseases results from the tripartite interaction of host x microbiota x pathogen (van Tol and Dimopoulos, 2016), and therefore an emergent property of the holobiont. The reason why the hologenome concept is needed is that it compels us to recognize that phenotypes, which may result from the interaction of many gene products, do not necessarily emerge from a single organism.
